# MISP Is Overexpressed in Intestinal Metaplasia and Gastric Cancer

**DOI:** 10.3390/curroncol31050210

**Published:** 2024-05-14

**Authors:** Tomás Vilarinho, Diana Pádua, Bruno Pereira, Patrícia Mesquita, Raquel Almeida

**Affiliations:** 1 i3S—Institute for Research and Innovation in Health, University of Porto, 4200-135 Porto, Portugal; tomas.castro@i3s.up.pt (T.V.); dpadua@ipatimup.pt (D.P.); bpereira@ipatimup.pt (B.P.); pmesquita@ipatimup.pt (P.M.); 2 IPATIMUP—Institute of Molecular Pathology and Immunology, University of Porto, 4200-465 Porto, Portugal; 3 Biology Department, Faculty of Sciences, University of Porto, 4169-007 Porto, Portugal

**Keywords:** MISP, gastric cancer, intestinal metaplasia

## Abstract

Gastric cancer is the fifth most common cancer and the fourth cause of global cancer mortality. The identification of new biomarkers and drug targets is crucial to allow the better prognosis and treatment of patients. The mitotic spindle positioning (MISP) protein has the function of correcting mitotic spindle positioning and centrosome clustering and has been implicated in the cytokinesis and migration of cancer cells. The goal of this work was to evaluate the expression and clinical relevance of MISP in gastric cancer. MISP expression was evaluated by immunohistochemistry in a single hospital series (n = 286) of gastric adenocarcinomas and compared with normal gastric mucosa and intestinal metaplasia, a preneoplastic lesion. MISP was detected on the membrane in 83% of the cases, being overexpressed in gastric cancer compared to normal gastric mucosa (n = 10). Its expression was negatively associated with diffuse and poorly cohesive types. On the other hand, it was strongly expressed in intestinal metaplasia where it was associated with MUC2 and CDX2 expression. Furthermore, when we silenced MISP in vitro, a significant decrease in the viability of gastric carcinoma cells was observed. In conclusion, MISP is overexpressed in gastric cancer, being associated with an intestinal phenotype in gastric carcinogenesis and having a role in cellular proliferation.

## 1. Introduction

Gastric cancer (GC) is the fifth most common cancer type and the fourth leading cause of global cancer mortality [1]. Patients are primarily treated with surgery, radiotherapy, and chemotherapy depending on the tumor stage and location [2], but the outcome of patients with GC remains heterogeneous, even within the same disease stage, and is often dismal. Thus, a better understanding of the disease is urgently needed to improve early diagnosis and treatment. The majority of GC are adenocarcinomas [3] that can be classified, according to Laurén’s classification system, as intestinal, diffuse and mixed types, depending on the tissue architecture and glandular patterns [4]. Diffuse-type tumors are recognized for their lack of cohesion, poor differentiation, and absence of gland formation. In contrast, intestinal-type tumors exhibit moderate-to-well-differentiated features and typically display a glandular structure. Mixed-type tumors, as the name suggests, present a mixture of characteristics from both the diffuse and intestinal types [4].

The intricate process of gastric carcinogenesis often starts with inflammation, frequently triggered by infection with *Helicobacter pylori*. This inflammatory response progresses through various stages, including multifocal atrophic gastritis, intestinal metaplasia (IM), dysplasia, and, ultimately, the development of cancer [5]. Among these stages, IM holds particular significance as it signifies a transition from a gastric to an intestinal differentiation profile. It is a prevalent precancerous condition in GC, being observed adjacent to more than 80% of GC cases, thereby carrying substantial clinical implications [6,7]. IM is characterized by the presence of specific markers indicative of intestinal differentiation, such as caudal-related homeobox transcription factor 2 (CDX2) and intestinal mucin 2 (MUC2) [8,9]. CDX2, a pivotal homeobox transcription factor crucial for intestinal differentiation [10,11], assumes a critical role in the early stages of gastric carcinogenesis by driving the initiation of IM [12,13,14,15]. Its expression serves as a specific biomarker, offering insights into the early phases of the gastric carcinogenic process.

The mitotic spindle positioning protein (MISP or C19orf21), a substrate of the Polo-like kinase 1 (PLK1), is involved in correcting the mitotic spindle positioning and centrosome clustering, being also involved in stress fiber and other thick actin filaments’ organization [16,17,18,19]. MISP is associated with the actin cytoskeleton and focal adhesions and has been functionally linked with cytokinesis and directed cancer cell migration [16,17]. The depletion of MISP in cancer cell lines causes mitotic arrest, impairs the proper mitotic spindle positioning and orientation, and reduces cell migration [17]. Interestingly, it has been shown to facilitate mitotic progression in cancer cells that harbor supernumerary centrosomes [17]. MISP operates downstream of ezrin to facilitate an optimal spindle positioning [19]. Ezrin, a critical cytoskeletal organizer, regulates interactions between the membrane and cytoskeleton, maintaining cell shape and structure and controlling cell adhesion, movement, and survival. Moreover, ezrin plays a pivotal role in regulating tumor metastasis by interacting with various binding proteins [20]. In pancreatic cancer, MISP has been associated with cell migration and epithelial–mesenchymal transition (EMT) [21]. Despite the links with these cancer cell features, the expression and function of MISP in cancer are largely unexplored.

Here, we sought to characterize the expression of MISP in GC and study its association with clinicopathological and differentiation features. We further analyzed the impact of downregulating MISP expression in two gastric carcinoma cell lines. In summary, our findings revealed that MISP was overexpressed in over 80% of GC cases, exhibiting strong expression in IM, where it was associated with the expression of MUC2 and CDX2, indicating a close association with an intestinal phenotype in gastric carcinogenesis. Additionally, in vitro silencing of MISP led to a significant decrease in the viability of gastric carcinoma cells.

## 2. Materials and Methods

### 2.1. Patient Cohorts

The GEPIA 2 database (http://gepia2.cancer-pku.cn/#index (accessed on 26 June 2023)), which combines gene expression data from TCGA and GTEx, was used to compare the expression of MISP in GC with normal gastric mucosa, using the information from the RNA-seq deposited in these databases. A second group of patients, comprising 286 individuals diagnosed with GC, was also used in this study. Histological specimens were obtained from surgical samples collected from consecutive cases of GC treated surgically between January 2008 and December 2014 at the Centro Hospitalar São João (CHSJ) in Porto, Portugal. Detailed records pertaining to tumor tissue, clinicopathological data, and follow-up information were available for all the patients included in this cohort. The utilization of retrospective samples, for which obtaining informed consent was not feasible, is permitted for research investigations by Portuguese law. Approval for conducting this study was granted by the ethics committee of the Centro Hospitalar São João under the reference number CES 122-15. Among the 286 patients, 107 individuals received chemotherapy, predominantly comprising platinum- and/or fluoropyrimidine-based regimens, with most treatments administered as adjuvant therapy following surgery. Additional clinical insights regarding this patient cohort can be found in studies authored by Mesquita et al. [22] and Lopes et al. [23], as well as in Table 1. In five cases, the examination extended beyond the tumors to include the study of normal gastric mucosa and areas of IM adjacent to the tumors.

### 2.2. Tissue Microarrays and Immunohistochemistry

Formalin-fixed, paraffin-embedded tumor tissue blocks from surgical specimens were utilized to create tissue microarrays (TMAs) for analysis. These TMAs were prepared by sectioning the tissue with a microtome (Microm HM 335 E) to a thickness of 4 µm. The expression of MISP was assessed using immunohistochemistry (IHC) staining, following the standardized protocols outlined in Mesquita et al. [22]. The staining procedure involved several steps: firstly, the tissues were deparaffinized and hydrated. Heat-induced epitope retrieval was performed using an IHC-Tek Epitope Retrieval Steamer Set, conducted for 40 min in 10 mM Tris-EDTA buffer at pH 9.0. To block endogenous peroxidase activity, the tissues were treated with 3% hydrogen peroxide for 10 min. The primary antibody used was a rabbit recombinant polyclonal MISP antibody (anti-MISP, Sigma-Aldrich, St. Louis, MO, USA, catalog number HPA062232) at a dilution of 1:500, incubated overnight at 4 °C in a humidified chamber. The detection of the antibody was achieved using the Dako REAL Envision Detection System Peroxidase/DAB+ (DAKO). Following antibody detection, sections were counterstained with hematoxylin, dehydrated, and mounted. To determine the MISP expression levels, the samples were categorized as MISP-low if they exhibited either a negative result for MISP staining or if MISP was present in less than 20% of malignant cells. Conversely, the samples were classified as MISP-high if MISP staining was observed in more than 20% of malignant cells. This classification was agreed upon by three independent observers. The images of the stained samples were captured using a Phenoimager^TM^ HT instrument (Akoya Biosciences, Marlborough, MA, USA) for further analysis.

### 2.3. Immunofluorescence

GC tissues were first deparaffinized and hydrated. Heat-induced epitope retrieval was carried out in an IHC-Tek Epitope Retrieval Steamer Set for 40 min in Tris-EDTA pH 9.0. After incubation with normal serum (1:5) for 30 min at room temperature, the tissues were incubated with the primary anti-MISP antibody (1:100 dilution, HPA062232, Sigma-Aldrich, Merck KGaA, Darmstadt, Germany) and the anti-CDX2 antibody (1:50 dilution, CDX2-88, Biogenex, Fremont, CA, USA) or with the anti-MUC2 antibody (1:50 dilution, CCP58, DAKO, Glostrup, Denmark) overnight at 4 °C. The tissues were incubated with secondary antibodies for 45 min in the dark at room temperature. The secondary antibody used for MISP was goat anti-rabbit labeled with Alexa Fluor 488 (diluted 1:100; A-11034, Thermo Fisher Scientific, Waltham, MA, USA), and the secondary antibody used for CDX2 and MUC2 was goat anti-mouse Alexa Fluor 594 (diluted 1:100; A-11032, Thermo Fisher Scientific, Waltham, MA, USA). The tissues were washed with PBS and incubated with DAPI (1 μg/mL) for 5 min at room temperature. Then, they were washed again with PBS, and the slides were assembled using the Vectashield mounting medium (Vector Laboratories, Burlingame, CA, USA). The images were obtained using a Zeiss Axio Imager Z1 microscope (Carl Zeiss, Oberkochen, Germany).

### 2.4. Cell Culture

Two human gastric carcinoma cell lines—AGS (ATCC—American Type Culture Collection, Manassas, VA, USA) and SNU638 (KCLB—Korean Cell Line Bank, Seoul, Republic of Korea)—were cultured in RPMI medium 1640 with 25 mM Hepes and GlutaMAX-1 (Gibco, Waltham, MA, USA) supplemented with 10% fetal bovine serum (FBS) (Biowest, Nuaillé, France) and maintained at 37 °C and 5% (*v/v*) CO_2_.

### 2.5. Transfection with esiRNAs

Cells (0.3 × 10^5^/well) were cultured in 24-well plates and allowed to attach overnight. The cells were transiently transfected with 200 nM of MISSION endonuclease-prepared siRNAs (esiRNAs) targeting human MISP (Sigma-Aldrich, Merck KGaA, Darmstadt, Germany) or MISSION esiRNA targeting Renilla luciferase (Sigma-Aldrich, Merck KGaA, Darmstadt, Germany), which was used as a negative control. The transfection was performed using Lipofectamine 2000 Thermo Fisher Scientific, Waltham, MA, USA) and serum-free Opti-MEM medium (Gibco, Waltham, MA, USA). After 24 h, the transfection medium was replaced with RPMI medium supplemented with 10% FBS. After another 24 h, cell viability was evaluated using the PrestoBlue Cell Viability Reagent 1× (Thermo Fisher Scientific, Waltham, MA, USA) according to the manufacturer’s instructions.

### 2.6. Western Blot

The cells underwent trypsinization, followed by washing with PBS, pelleting, and resuspension in cold RIPA lysis buffer. This buffer composition included 50 mM Tris-HCl (pH = 7.4), 150 mM NaCl, 2 mM EDTA, 1% NP-40, and 0.1% SDS, supplemented with a complete protease inhibitor cocktail (Roche, Basel, Switzerland), 1 mM PMSF, and 1 mM Na_3_VO_4_ to ensure protein stability and prevent degradation. To assess the protein expression levels, 40 μg of total protein extract was subjected to separation via SDS-PAGE, employing the Precision Plus Protein Standard Dual Color (Bio-Rad, Hercules, CA, USA) as a protein marker. Following electrophoresis, the proteins were transferred onto a nitrocellulose membrane (Amersham, GE Healthcare, Chicago, IL, USA) for subsequent immunoblotting. The membranes were initially blocked with 5% skimmed milk in TBS-1% Tween-20 (Sigma-Aldrich, Merck KGaA, Darmstadt, Germany) for 1 h at room temperature to prevent nonspecific binding. Subsequently, they were probed overnight at 4 °C with primary antibodies targeting MISP (dilution 1:1000, HPA062232, Sigma-Aldrich, Merck KGaA, Darmstadt, Germany) and β-actin (dilution 1:2000; Santa Cruz Biotechnology, Dallas, TX, USA) to serve as an internal control. After thorough washing with TBS-1% Tween-20 to remove unbound antibodies, the membranes were then exposed to the respective HRP-conjugated secondary antibodies: goat anti-rabbit IgG (diluted 1:5000; Cell Signaling Technology, Danvers, MA, USA) for MISP or goat anti-mouse IgG (diluted 1:1500; Santa Cruz Biotechnology, Dallas, TX, USA) for β-actin. Signal detection was achieved by employing an ECL detection kit (Amersham, GE Healthcare, Chicago, IL, USA). The expression levels of MISP were normalized against β-actin to account for loading variations.

### 2.7. Statistical Analysis

Our objective was to assess the correlation between MISP expression status and the clinicopathological characteristics of the tumors, as outlined in Table 1. To achieve this, we employed various statistical tests tailored to the specific parameters under investigation. To compare patient age, we utilized Student’s *t*-test. Fisher’s exact test (two-sided) was employed to analyze gender distribution and treatment options. For evaluating tumor stage, Laurén’s classification and WHO classification, we employed the Chi-square test (χ^2^). The association of MISP with patient survival (overall and disease-free survival) was analyzed according to the Kaplan–Meier method, and differences between survival distributions were assessed with the log-rank test. Differences were considered statistically significant when the *p*-value was less than 0.05.

In the cell viability assays, the results were presented as the mean ± standard deviation (SD) derived from a minimum of three independent experiments. Statistical significance was determined using the unpaired two-tailed *t*-test, which compares the means of two independent groups. Differences were considered statistically significant when the *p*-value was less than 0.05. This stringent criterion helped to ensure the robustness and validity of our findings.

## 3. Results

### 3.1. MISP Is Overexpressed in GC

**Figure 1:**
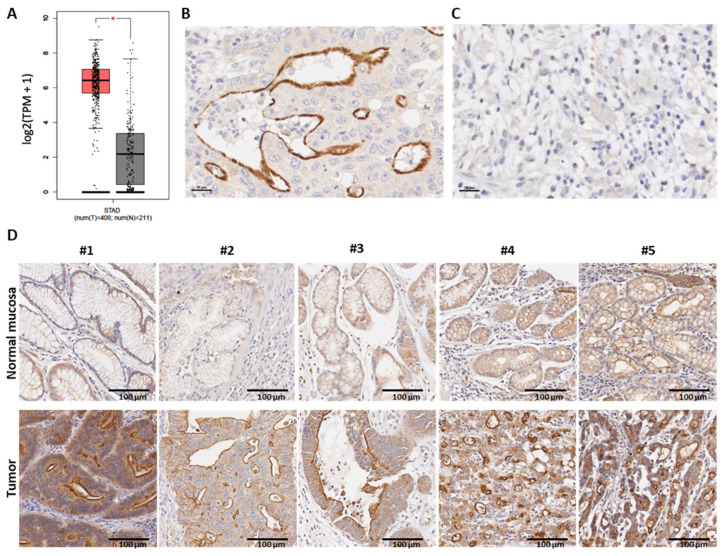
Expression levels of MISP in GC. (**A**) Analysis of MISP RNA expression in gastric tumors (n = 408, in red) compared with normal gastric mucosa (n = 211, in grey) based on TCGA and GTEx data (TPM: transcript per kilobase million; STAD: stomach adenocarcinoma; *: statistically significant difference). MISP protein expression in the apical membrane (**B**) in a positive case compared to (**C**) a negative case, and (**D**) strong MISP expression in GC, compared to the gastric mucosa, where it is weak or absent. In (**B**,**C**), the scale bar presented is 20 µm while in (**C**,**D**) is 100 µm.

We started by characterizing MISP expression in normal gastric mucosa and GC using RNA-seq data deposited in the databases TCGA and GTex. This analysis showed that MISP was overexpressed in GC when compared to normal gastric mucosa (Figure 1A). To validate these results in another patient cohort and at the protein level, we characterized the expression of MISP in a series of 286 GC cases from all stages and categorized the expression in two groups, high and low (includes negative cases), as described in the Material and Methods Section. High levels of MISP expression were observed in 236 cases (82.5%), and expression was low in 50 cases (17.5%). MISP expression was detected on the apical membrane, as it can be seen in the representative image of a positive case shown in Figure 1B. Figure 1C showcases a negative case. Next, we compared MISP expression in GC and the adjacent gastric mucosa in five cases. It was clear that MISP expression was stronger in GC compared to the adjacent gastric mucosa where it was expressed at a very low level (Figure 1D). Thus, considering that the vast majority of GC cases expressed MISP, the results obtained in both GC patient cohorts clearly indicate that MISP is overexpressed in GC.

### 3.2. MISP Is Associated with Intestinal-Type GC

The clinicopathological characteristics of the 286 GC cases and their relationship with MISP expression are outlined in Table 1. Notably, elevated levels of MISP were found to be significantly correlated with specific tumor classifications according to the Laurén classification system. In particular, a high expression of MISP was observed in tumors categorized as intestinal, noted in 86.2% of the cases, as well as in mixed-type tumors, observed in 86.1% of cases (*p* < 0.001). Diffuse-type tumors were only 12.2% of the cases, and only 57.1% of them expressed high levels of MISP. This patient series was also characterized regarding patient survival [22,23], but MISP did not show value as a prognostic marker.

### 3.3. MISP Is Overexpressed in IM

**Figure 2:**
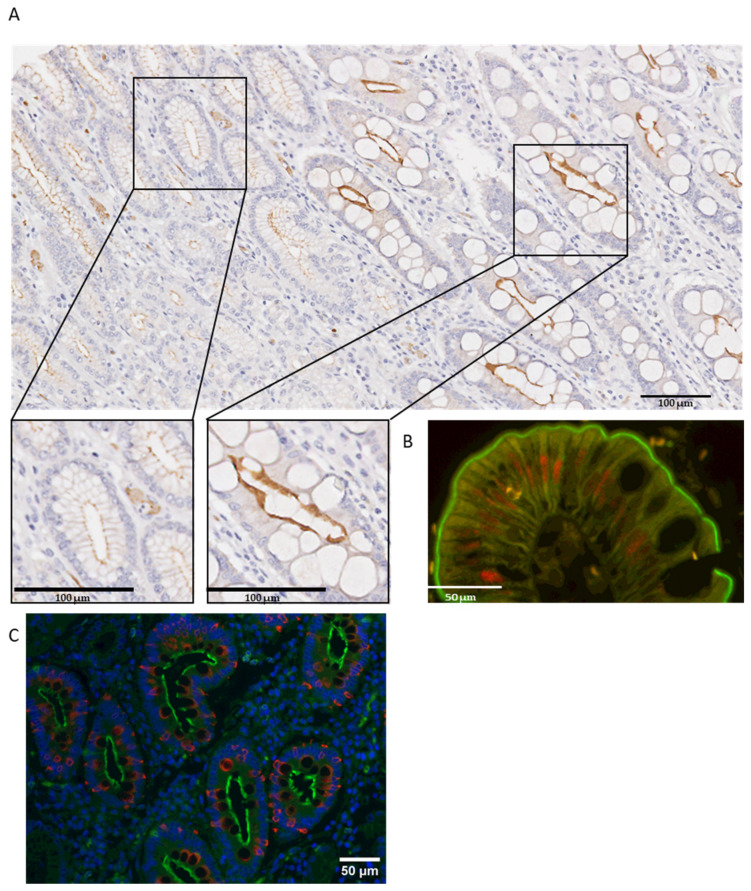
Expression levels of MISP in GC. (**A**) Higher levels of MISP in IM, when compared with normal gastric mucosa, and (**B**) immunofluorescence reveals double-staining of MISP (in green) and CDX2 (in red) and (**C**) MISP (in green) and MUC2 (in red) in IM.

We also analyzed MISP expression in the preneoplastic lesion IM, which is frequently present in areas adjacent to GC. MISP was strongly expressed in IM, compared to the adjacent gastric mucosa (Figure 2A). To confirm the association of MISP with IM, we performed double-staining of MISP with CDX2 or MUC2, which are well-established markers of intestinal differentiation. The co-localization of MISP and CDX2 (Figure 2B) or MISP and MUC2 (Figure 2C) in the same glands was evident. CDX2 was expressed in the nuclei of the cells, MUC2 appeared in the goblet cells, and MISP was expressed in the apical membrane of the same cells.

### 3.4. MISP Downregulation Impacts GC Cell Viability In Vitro

**Figure 3:**
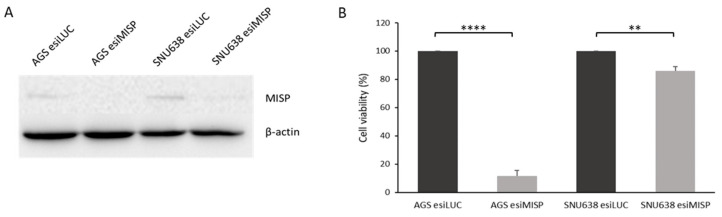
Cell viability after the silencing of MISP in gastric carcinoma cells. (**A**) MISP expression was evaluated by Western blot in AGS and SNU638 cells transfected with an esiRNA targeting human MISP. Cells transfected with esiRNA targeting Renilla luciferase (LUC) were used as a negative control. β-actin was used as an internal control. The uncropped blots are shown in the Supplementary Materials. (**B**) Cell viability was assessed in the same cells. The results are the mean ± SD of three independent experiments. Significant differences (** *p* ≤ 0.01; **** *p* ≤ 0.0001).

Finally, to study the relevance of MISP expression in GC, we used esiRNAs to downregulate its expression in two GC cell lines (AGS and SNU638) and assessed the impact on cellular viability. The results showed that MISP downregulation (Figure 3A) led to a decreased cell number 48 h after transfection, especially in AGS cells, where only 11.5% of the cells survived (Figure 3B).

## 4. Discussion

MISP is a protein involved in centrosome clustering and mitotic spindle orientation. These processes are essential for mitotic progression and cell motility, which are hallmarks of cancer cells. Nevertheless, the role of MISP in cancer is far from being understood. In this study, we assessed the expression and relevance of MISP in GC and showed, for the first time, that MISP is expressed in the vast majority of GC cases and that its expression is increased when compared with normal gastric mucosa. MISP was first identified in a genome-wide RNAi screen as a protein involved in centrosomal clustering in cancer cells with extra centrosomes [17]. Centrosomal clustering allows cancer cells with supernumerary centrosomes to successfully divide in a bipolar manner, thus avoiding lethal multipolar divisions. We can speculate that MISP expression in cancer is important to overcome chromosome number aberrations and allow mitosis to occur. On the other hand, it was found that directed cell migration, which is a focal adhesion and microtubule-dependent process, also relied on MISP’s correct expression and cellular localization [17]. In another study, the in vitro knockdown of MISP in cholangiocarcinoma cells resulted in reduced trans-lymphatic endothelial migration and impaired wound healing, alongside alterations in focal adhesions [24]. Thus, it might be worth to further explore MISP function in cancer cell behavior and aggressiveness. When we downregulated MISP expression in GC cell lines, the number of cells was decreased compared with the controls, suggesting either cell death or a slower proliferation rate, as it has been observed that MISP depletion leads to an impaired metaphase-to-anaphase transition [16]. Concordantly, reducing MISP expression in pancreatic ductal adenocarcinoma cells results in inhibited cell proliferation [21]. Similarly, in human colorectal cancer (CRC) cell lines, the knockdown of MISP significantly diminishes the colony-forming ability, suggesting that MISP promotes CRC cell proliferation [25]. These findings parallel the outcomes of the present study, where decreased MISP expression correlates with reduced cell viability/proliferation in GC cells.

Regarding the molecular mechanisms involving MISP signaling in cancer, particularly in the context of pancreatic cancer, research indicates that the depletion of MISP leads to an increase in the accumulation ofthe IQ Motif Containing GTPase Activating Protein 1 (IQGAP1) at the cell cortex, concomitant with a reduction in active Cell Division Cycle 42 (Cdc42) levels. Notably, this effect can be reversed by overexpressing IQGAP1 [21]. This finding holds significance due to the pivotal role of activated Cdc42 in cellular processes such as pseudopodia elongation, modulation of the actin cytoskeleton, and polarization of epithelial cells. Accordingly, Vodicska et al. [26] demonstrated that MISP plays a pivotal role in regulating the IQGAP1/Cdc42 complex, thereby collectively coordinating spindle orientation and mitotic progression. MISP utilizes IQGAP1 as a downstream regulatory molecule for astral microtubule dynamics and the localization of the dynactin subunit p150 (glued). These authors also proposed that these effects could be attributed to the maintenance of IQGAP1 in an open and active form, capable of binding to Cdc42, which is facilitated by the interaction with MISP [26]. These cellular events collectively contribute to enhanced cell migration, invasion, and, ultimately, the progression of cancer. Therefore, the observed interaction between MISP, IQGAP1, and Cdc42 sheds light on potential mechanisms underlying cancer progression, highlighting promising avenues for further investigation and therapeutic intervention. Furthermore, Wang et al. showed that M2 macrophages promote the immune escape of hepatocellular carcinoma by upregulating PD-L1 through the MISP/IQGAP1/STAT3 axis [27]. In the context of GC, Shi et al. showed that MISP, through PLK1-mediated phosphorylation, is part of a signaling pathway that renders GC cells sensitive to trastuzumab in a HER2-positive setting [28].

Another major finding of our study was the association of MISP expression with the intestinal phenotype, evidenced by a higher expression of MISP in the preneoplastic lesion IM and, also, in intestinal-type gastric adenocarcinoma. An intestinal differentiation hallmark of these lesions is the expression of CDX2 and MUC2 [8,9,10,11]. Our findings demonstrate that MISP exhibits co-expression with CDX2 and MUC2 in IM, indicating its association with intestinal differentiation at an early stage in the carcinogenic pathway. Our observations provide significant insights into the spatial distribution and co-localization of MISP with CDX2 and MUC2. This co-expression pattern underscores the potential role of MISP in the pathogenesis of GC and highlights its relevance as a candidate biomarker for early detection and therapeutic targeting strategies. The tissue specificity of MISP expression has not been extensively explored, but, indeed, Morales et al. showed that MISP is expressed in the intestinal brush border and has the function of F-actin bundling, which is critical in supporting microvilli in small intestinal tissues [29]. Another report showed that MISP is localized in the apical membrane in the colon, in accordance with our observations in gastric glands, IM, and GC [27]. Moreover, they showed that, in a Dextran Sulfate Sodium (DSS)-induced colitis mouse model, MISP deficiency led to exacerbated inflammation and decreased cell proliferation in the crypts, which revealed its role in protecting against colitis [30]. These discoveries suggest that MISP could be involved in colon recovery following inflammation, owing to its anti-inflammatory and proliferative properties. Such indications hint at the potential of MISP as a novel therapeutic target for inflammatory bowel disease (IBD). In a follow-up investigation led by the same researchers, it was revealed, in the same mouse model, a notable reduction in CRC development in the context of colitis. This discovery implies that MISP might contribute as a potential risk factor for CRC through its interaction with Opa Interacting Protein 5 (OIP5), forming a complex which triggers the activation of the JAK2-STAT3 (Janus kinase 2-Signal transducer and activator of transcription 3) signaling pathway [25].

## 5. Conclusions

In conclusion, MISP is overexpressed in GC and associates with intestinal differentiation, and its downregulation leads to decreased cell proliferation in GC cell lines. Thus, MISP may play a relevant role in GC cell survival and proliferation, which deserves further clarification.

## Figures and Tables

**Table 1:** Clinicopathological data and MISP expression in 286 patients with GC.

	All Cases		MISP High		MISP Low		*p*
	*n*	%	*n*	%	*n*	%	
**Patients**	**286**		**236**	82.5	**50**	17.5	
**Age**							
Mean ± SD	**67.9 ± 11.9**		**68.9 ± 11.4**		**63.4 ± 13.4**		0.196
Range	**32–95**		**33–95**		**32–87**		
**Gender**							
Female	**121**	42.3	**101**	83.5	**20**	16.5	0.755
Male	**165**	57.7	**135**	81.8	**30**	18.2	
**Stage**							0.211
I	**94**	32.9	**78**	83.0	**16**	17.0	
II	**73**	25.5	**65**	89.0	**8**	11.0	
II	**64**	22.4	**51**	79.7	**13**	20.3	
IV	**55**	19.2	**42**	76.4	**13**	23.6	
**Laurén classification**							
Intestinal	**138**	48.3	**119**	86.2	**19**	13.8	<0.001 *
Diffuse	**35**	12.2	**20**	57.1	**15**	42.9	
Mixed	**72**	25.2	**62**	86.1	**10**	13.9	
Unclassified	**41**	14.3					
**WHO classification**							
Mucinous	**6**	2.1	**5**	83.3	**1**	16.7	0.018 *
Papillary	**1**	0.4	**1**	100.0	**0**	0.0	
Poorly cohesive	**30**	10.5	**19**	63.3	**11**	36.7	
Tubular	**127**	44.3	**113**	89.0	**14**	11.0	
Other types	**122**	42.7	**98**	80.3	**24**	19.7	
**Chemotherapy**							0.025 *
No	**177**	61.9	**153**	86.4	**24**	13.6	
Yes	**107**	37.4	**81**	75.7	**26**	24.3	
ND	**2**	0.7					

Notes: *p* values (statistical significance threshold < 0.05) are obtained using Student’s *t* test for the continuous variable, Fisher’s exact test (two-sided), and the Chi-square (χ^2^) test for categorical variables; * comparisons with *p* < 0.05. ND = not determined; SD = standard deviation.

## Data Availability

The datasets used and/or analyzed during the current study are available from the corresponding author upon reasonable request.

## Supplementary Materials

The following supporting information can be downloaded at: https://www.mdpi.com/article/10.3390/curroncol31050210/s1, Full pictures of the Western blots.
