# Supplementary figures and images for: MISP Is Overexpressed in Intestinal Metaplasia and Gastric Cancer
Source: Curr Oncol. 2024 May 14;31(5):2769–79. doi: 10.3390/curroncol31050210 (PMC11120023; doi:10.3390/curroncol31050210)

## Slide 1
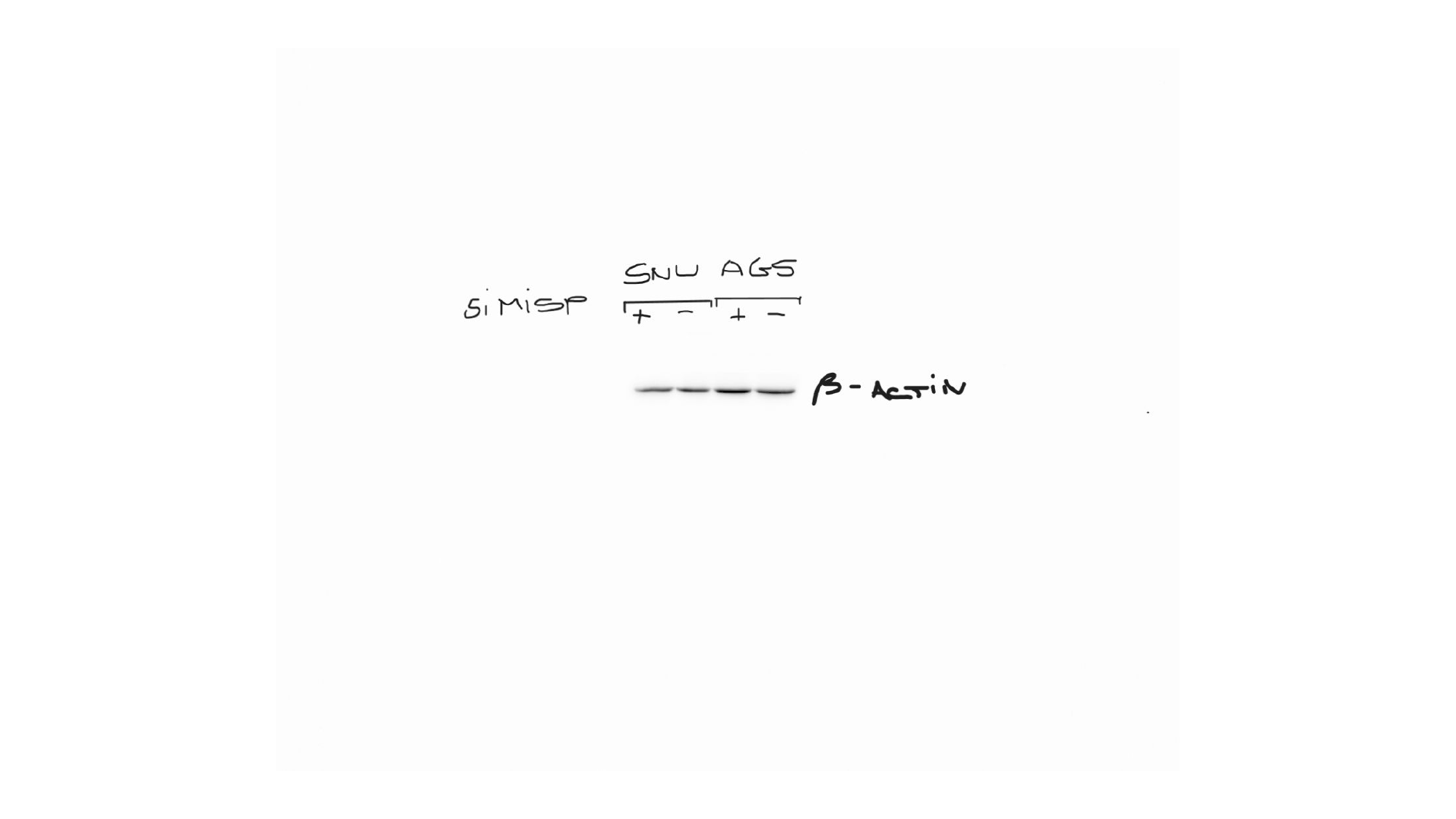

## Slide 2
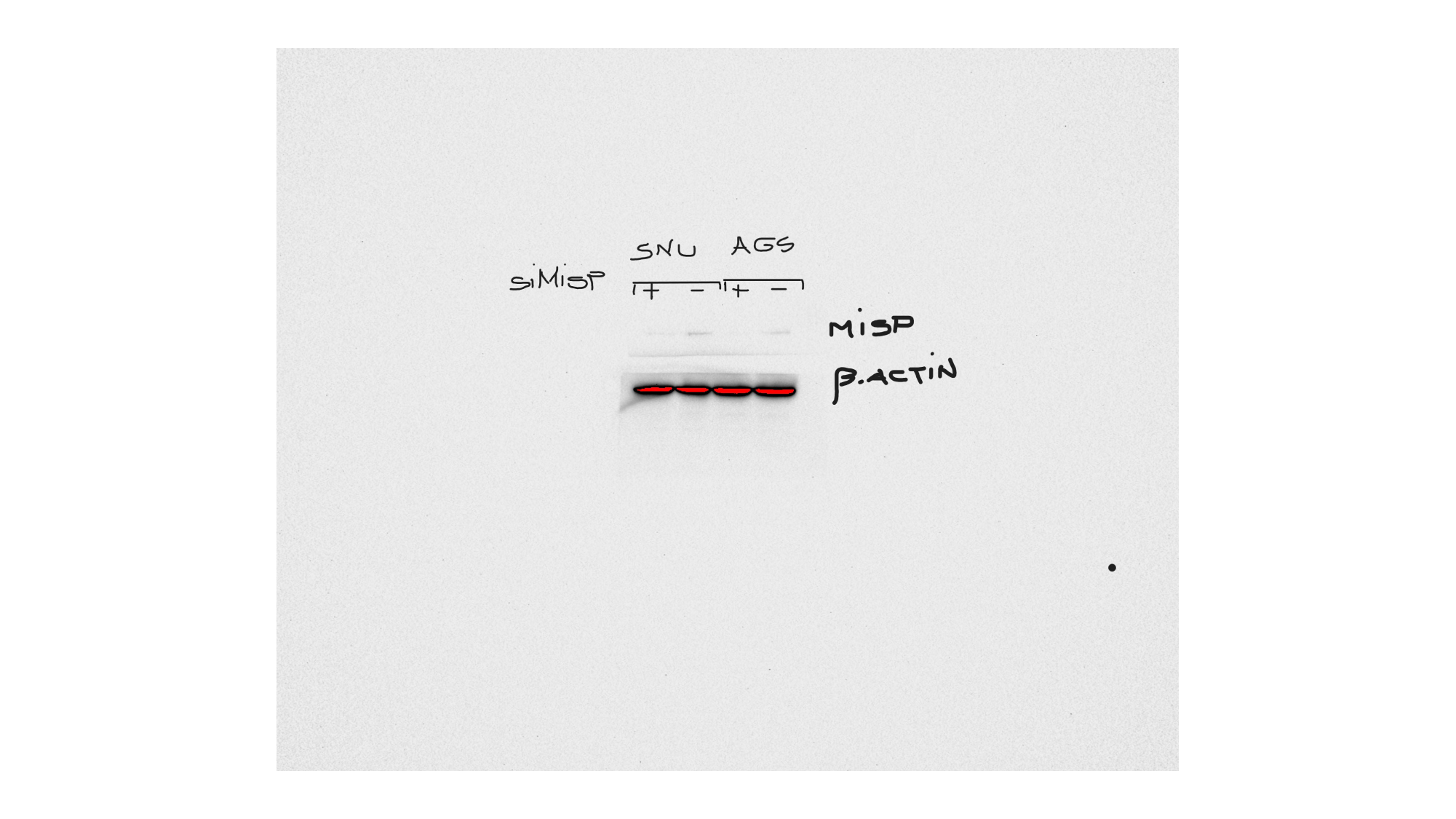

Supplement: Supplementary file 1 [file curroncol-31-00210-s001.zip › curroncol-2971732-supplementary.pptx]
